# Pomalidomide Ameliorates H_2_O_2_-Induced Oxidative Stress Injury and Cell Death in Rat Primary Cortical Neuronal Cultures by Inducing Anti-Oxidative and Anti-Apoptosis Effects

**DOI:** 10.3390/ijms19103252

**Published:** 2018-10-19

**Authors:** Yan-Rou Tsai, Cheng-Fu Chang, Jing-Huei Lai, John Chung-Che Wu, Yen-Hua Chen, Shuo-Jhen Kang, Barry J. Hoffer, David Tweedie, Weiming Luo, Nigel H. Greig, Yung-Hsiao Chiang, Kai-Yun Chen

**Affiliations:** 1 Ph.D. Program for Neural Regenerative Medicine, College of Medical Science and Technology, Taipei Medical University and National Health Research Institutes, Taipei 11031, Taiwan; aggytsai@hotmail.com; 2 Center for Neurotrauma and Neuroregeneration, Taipei Medical University, Taipei 11031, Taiwan; chang3@ms3.hinet.net (C.-F.C.); m105095006@tmu.edu.tw (J.-H.L.); dr.jcwu@gmail.com (J.C.-C.W.); swallows3366@gmail.com (Y.-H.C.); terbiun@gmail.com (S.-J.K.); bhoffer@intra.nida.nih.gov (B.J.H.); 3 Department of Neurosurgery, Taipei City Hospital, Zhongxiao Branch, Taipei 11556, Taiwan; 4 Department of Surgery, College of Medicine, Taipei Medical University, Taipei 11031, Taiwan; 5 Department of Neurosurgery, Taipei Medical University Hospital, Taipei 11031, Taiwan; 6 Department of Neurosurgery, Case Western Reserve University, School of Medicine, Cleveland, OH 44106, USA; 7 Drug Design & Development Section, Translational Gerontology Branch, Intramural Research Program, National Institute on Aging, National Institutes of Health, Baltimore, MD 20892, USA; tweedieda@grc.nia.nih.gov (D.T.); luowe@grc.nia.nih.gov (W.L.); greign@grc.nia.nih.gov (N.H.G.)

**Keywords:** pomalidomide, neuroprotection, oxidative stress, ischemia, stroke, neurodegeneration

## Abstract

Due to its high oxygen demand and abundance of peroxidation-susceptible lipid cells, the brain is particularly vulnerable to oxidative stress. Induced by a redox state imbalance involving either excessive generation of reactive oxygen species (ROS) or dysfunction of the antioxidant system, oxidative stress plays a central role in a common pathophysiology that underpins neuronal cell death in acute neurological disorders epitomized by stroke and chronic ones such as Alzheimer’s disease. After cerebral ischemia, for example, inflammation bears a key responsibility in the development of permanent neurological damage. ROS are involved in the mechanism of post-ischemic inflammation. The activation of several inflammatory enzymes produces ROS, which subsequently suppress mitochondrial activity, leading to further tissue damage. Pomalidomide (POM) is a clinically available immunomodulatory and anti-inflammatory agent. Using H_2_O_2_-treated rat primary cortical neuronal cultures, we found POM displayed neuroprotective effects against oxidative stress and cell death that associated with changes in the nuclear factor erythroid derived 2/superoxide dismutase 2/catalase signaling pathway. POM also suppressed nuclear factor kappa-light-chain-enhancer (NF-κB) levels and significantly mitigated cortical neuronal apoptosis by regulating Bax, Cytochrome c and Poly (ADP-ribose) polymerase. In summary, POM exerted neuroprotective effects via its anti-oxidative and anti-inflammatory actions against H_2_O_2_-induced injury. POM consequently represents a potential therapeutic agent against brain damage and related disorders and warrants further evaluation.

## 1. Introduction

Neurological diseases account for the world’s largest cause of disability, consequent to 250.7 million disability-adjusted life years (DALYs) that were lost during 2015 together with 9.4 million deaths [1]. Globally, stroke accounts for the largest proportion of these DALYs, at 47.3% of the total (some 118.6 million) and deaths, at 67.3% (6.3 million) [1]. Indeed, stroke is the second major cause of death and the leading cause of long-term neurological disability worldwide [2], with Alzheimer’s disease and related dementias representing the second largest contributor to deaths and DALYs from neurological disorders. There is a strong epidemiological association between vascular factors predisposing to cerebrovascular disease or stroke and AD [3] and many of the pathophysiological underpinnings are common across these disorders, likely accounting for the 60–90% of AD patients that show cerebrovascular pathology at autopsy. Coexistence of stroke and AD are at far greater than chance alone levels [4,5,6].

Ischemic stroke represents approximately 80 to 85% of all strokes and has been the target of most drug trials [7,8,9]. After cerebral ischemia, inflammation plays a key role in the development of permanent neurological damage [10] and is similarly evident in the AD brain [11,12]. Brain ischemia produces superoxide through xanthine oxidase and leakage from the mitochondrial electron transport chain [13]. Superoxide is the primary radical from which hydrogen peroxide (H_2_O_2_) is formed. In turn, H_2_O_2_ is the source of the hydroxyl radical (OH), which readily crosses the cell membrane [14,15]. Free radicals manifest a series of cellular effects including enzymatic inactivation, protein denaturation, cytoskeletal and DNA injury, lipid peroxidation and chemotaxis. Severe oxidative stress results in cell death through necrosis, while moderate oxidation gives rise to apoptosis [16,17,18]. Mitochondrial function is impaired by free radical-mediated breakdown of the inner mitochondrial membrane and the oxidation of proteins that mediate electron transport, H^+^ extrusion and adenosine triphosphate (ATP) production. As a result, Cytochrome c is released from the mitochondria and, following its binding to apoptotic protease activating factor-1 (Apaf-1) and formation of an apoptosome with Caspase-9, ensuing active caspase-3 cleavage impacts the downstream protein poly (ADP-ribose) polymerase (PARP) [19]. Substrate cleavage causes DNA injury and subsequently leads to apoptotic cell death [15].

Pomalidomide (POM) is a third-generation derivative of thalidomide. Thalidomide and its analogs (lenalidomide and POM) are immunomodulatory drugs that display potent biological effects on cytokine- and cell-mediated responses [20,21,22]. A recent study reported that POM was potently anti-inflammatory in embryonic and in vitro assays at significantly lower concentrations than the parent compounds, thalidomide and lenalidomide [23]. Moreover, as an immune modulator, POM has less adverse effects of teratogenic, anti-angiogenic and neurotoxic properties [23,24]. In this current paper, we demonstrate that POM prevented H_2_O_2_-induced oxidative stress injury in rat primary cortical neuronal cultures by inducing anti-oxidative and anti-apoptosis effects and preventing neuronal cell death. These anti-oxidative effects correlated with the activation of the nuclear factor erythroid derived 2 (Nrf2)/superoxide dismutase 2 (SOD2)/catalase signaling pathway, whereas the anti-apoptosis effects were associated with declines in the expression of BAX, Cytochrome c and PARP. Together, our cellular studies indicate that POM provides neuroprotective actions with potential in brain injury and warrants in vivo efficacy and safety evaluation as a new treatment strategy.

## 2. Results

The POM doses 50 nM and 500 nM were evaluated in our studies, which are in line with those reported in the literature [13]. Our focus was specifically on the POM 50 nM concentration. This is equivalent to a cellular dose of 13.65 ng/mL which is in accord with plasma levels attainable in humans (13 ng/mL) after a 2 mg dose [25] and hence can be considered readily achievable after a routine daily dose of 4 mg POM or a single higher dose [13].

### 2.1. Protective Effects of POM Against H_2_O_2_-Induced Cell Damage in Primary Cultures of Rat Cortical Neurons

**Figure 1:**
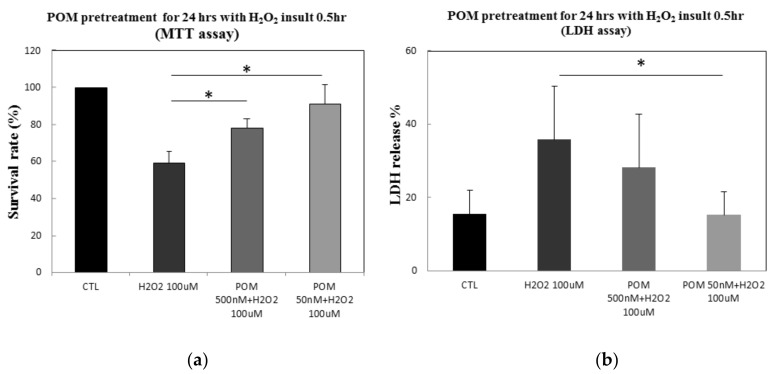
Effects of pomalidomide (POM) on H_2_O_2_-induced cellular death and cytotoxicity. (**a**) The MTT cell viability assay. The percentage of cell death was quantified by normalization of all values to the control (CTL) group (=100%). (**b**) The lactate dehydrogenase (LDH) release assay. Data are indicated as the percentage of LDH release of the injury group (H_2_O_2_-induced group). Compared to the control group, H_2_O_2_ alone (100 µM) induced a significant reduction in survival (MTT assay, *p* ≤ 0.05) and rise in LDH levels (*p* ≤ 0.05). Both were significantly mitigated by POM (* *p* < 0.05 versus 100 µM H_2_O_2_ alone challenged group). *Bars* represent mean values ± standard error of the mean (*n* = 4).

In order to assess the protective effects of POM under conditions similar to oxidative stress after brain ischemia, primary rat cortical neurons were pretreated with 500 nM or 50 nM of POM for 24 h and subsequently exposed to 100 µM of H_2_O_2_ for 0.5 h, in accord with other studies [26,27,28]. Exposure to H_2_O_2_ killed approximately 40% of the cells as compared to controls (*p* ≤ 0.05). Importantly, POM at 500 nM and 50 nM significantly increased cell viability by approximately 19.1% and 32.1%, respectively (Figure 1a).

The leakage of LDH is a well-established marker of injury of the cellular membrane [26,29,30]. After cells were exposed to 100 µM of H_2_O_2_ for 0.5 h, LDH release significantly increased (*p* ≤ 0.05). POM at 500 and 50 nM suppressed this H_2_O_2_-induced LDH release (Figure 1b), with the latter reaching statistical significance. These results indicate a protective effect of POM against H_2_O_2_-induced cellular death and cytotoxicity.

### 2.2. Pomalidomide Protects against H_2_O_2_-Induced Mitochondrial Superoxide Production and H_2_O_2_-Induced Cellular Apoptosis

**Figure 2:**
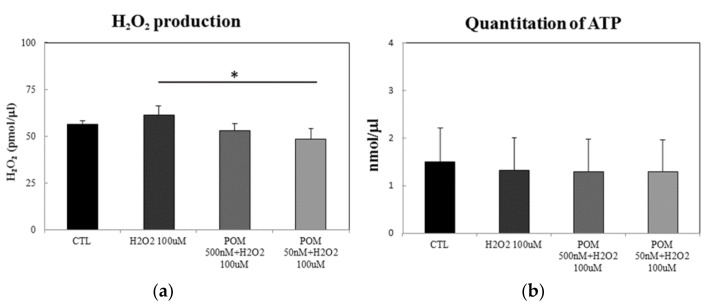
Effects of pomalidomide (POM) on H_2_O_2_-induced oxidative stress and adenosine triphosphate (ATP) levels. (**a**) The OxiRed Probe reacts with H_2_O_2_ to produce red fluorescence (Ex/Em = 535/587 nm). The results represent the mean ± standard error of the mean (SEM, *n* = 3). There was a statistically insignificant trend of elevated H_2_O_2_ amount in media in the H_2_O_2_ alone challenged group. Addition of POM 50 nM reversed this, with * *p* < 0.05 versus 100 µM H_2_O_2_ alone treated group. (**b**) The ATP assay kit utilizes the phosphorylation of glycerol to generate a product and is quantified by fluorometric method (Ex/Em = 535/587 nm). There were no significant differences among groups (mean ± SEM, *n* = 3).

To further explore the biological significance of secreted H_2_O_2_, we analyzed the levels of H_2_O_2_ in media from all four experimental groups. When primary rat cortical neurons were exposed to 100 µM of H_2_O_2_ for 0.5 h, we observed an increased production of H_2_O_2_ in the media. At 50 nM, POM significantly decreased the level of H_2_O_2_ production (Figure 2a). POM 500 nM demonstrated a trend to lower H_2_O_2_ generation that failed to reach statistical significance (*p* = 0.15). Hence, pretreatment with the lower dose of POM could reduce the impact of H_2_O_2_ in vitro.

Accumulation of ROS within the mitochondria hampers the functionality of this organelle, which could potentially lead to a decrease in ATP concentration. To evaluate this, intracellular ATP levels were examined in primary cortical neurons pretreated with POM and following injury with 100 µM of H_2_O_2_. After 0.5 h of H_2_O_2_ insult, intracellular ATP levels were lower across all injured groups with and without POM 50 and 500 nM; however, these differences did not reach statistical significance (Figure 2b).

**Figure 3:**
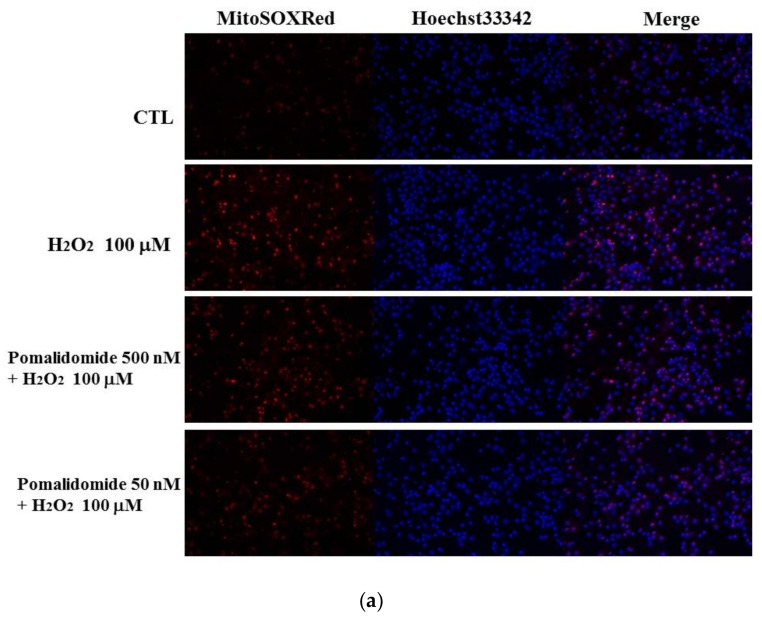

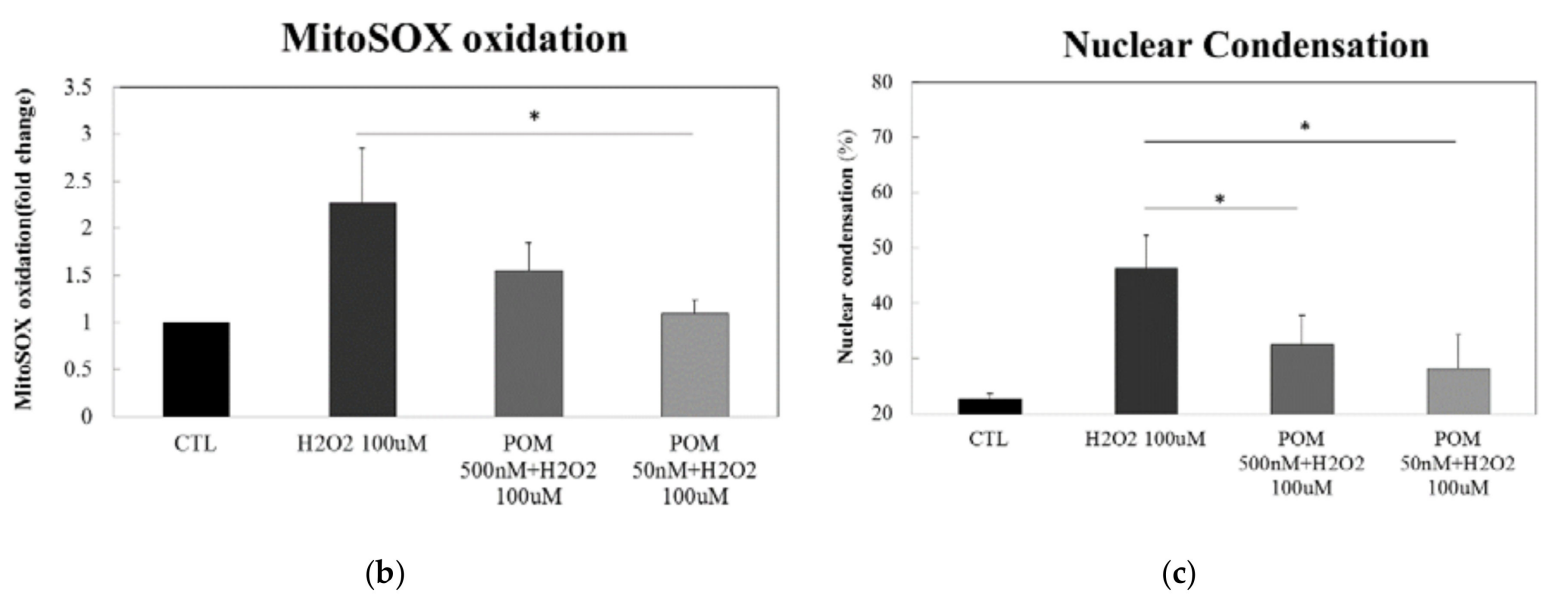
Detection of mitochondrial-specific superoxide in H_2_O_2_-induced oxidative stress. Cells were pretreated with POM for 24 h then exposed to 100 µM H_2_O_2_ for 0.5 h. Subsequently, (**a**) cells were labeled with mitochondrial-specific superoxide, detected using fluorogenic MitoSOX™ dye (red fluorescence, 1st panel), Hoechst 33342 (blue fluorescence, 2nd panel); merged (3rd panel) at 200× (magnification). (**b**) The fluorescence intensity fold change of MitoSOX™. The fluorescence intensity was quantified and normalized to the number of cells. Data are presented as the fold change relative to the control (CTL) group. (**c**) The nuclear condensation of Hoechst 33342, histogram showing the percentage of apoptotic cells (featured by nuclear condensation) in the cell population. Comparing the control group (CTL) to the H_2_O_2_ alone challenged group, both the Hoechst 33342 stain and MitoSOX™ Red stain are significantly different (*p* < 0.05). Notably, POM significantly mitigated H_2_O_2_-induced changes, with * *p* < 0.05 versus the 100 µM H_2_O_2_ alone challenged group. Data are representative of three independent experiments. Bars represent mean values ± SEM (*n* = 3).

Cellular production of ROS and related oxidants were measured by using molecular probes that become fluorescent in response to ROS and oxidants [31]. Cells were labeled with the mitochondrial-specific superoxide molecular probe MitoSox™ Red in combination with Hoechst 33342 blue fluorescence dye. Consistent with our other in vitro findings, after 0.5 h of H_2_O_2_ exposure, cortical neurons with the superoxide signal, detected by MitoSOX™ Red, had significantly enhanced fluorescence intensity (Figure 3a). Both POM pretreatment groups displayed a suppressed production of superoxide, shown by reduced MitoSOX™ Red fluorescence intensity (Figure 3b), that reached statistical significance for POM 50 nM.

We additionally appraised the fluorescence micrographs with Hoechst 33342 staining. This evaluation indicated the presence of apoptotic morphological features within the cells exposed to H_2_O_2_ [31,32], such as nuclear shrinkage, chromatin condensation and formation of apoptotic bodies (Figure 3a). Notably, pretreatment with 500 nM and 50 nM of POM significantly decreased the number of apoptotic cells, characterized by nuclear condensation, compared with the H_2_O_2_-exposed group (Figure 3c). Together, these results suggest that POM may protect against H_2_O_2_-induced mitochondrial superoxide production and H_2_O_2_-induced cell apoptosis.

### 2.3. Protein Expression of ROS Defense System Enzymes and Anti-Inflammation Responses

**Figure 4:**
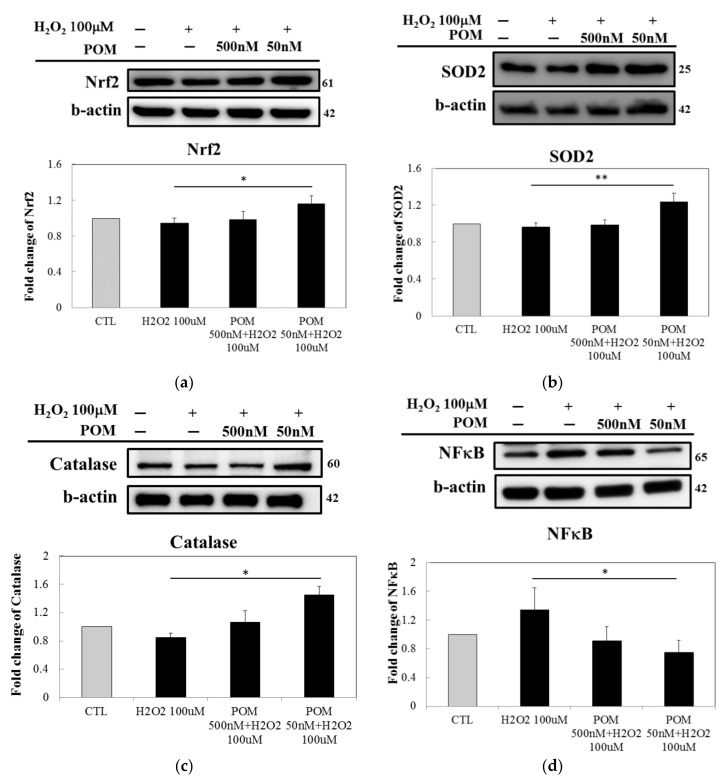
Pomalidomide (POM) activates Nrf2, which involves antioxidant enzymes SOD2 and Catalase and decreases NF-κB that is known in many cell types as a sensor for oxidative stress. In relation to the expression of antioxidant enzymes, there was no significant difference between the control (CTL) group and H_2_O_2_ alone challenged group. Notably, however, POM-pretreatment (50 nM) significantly enhanced the generation of antioxidant enzymes. As to H_2_O_2_-induced actions on the levels of NF-κB, whereas H_2_O_2_ alone mildly elevated NF-κB levels, POM-pretreatment (50 nM) markedly reduced NF-κB. Protein levels of Nrf2 (**a**), SOD2 (**b**), Catalase (**c**) and NF-κB (**d**) in primary rat cortical neurons were measured by Western blot analysis. β-actin served as a control. Bars represent mean values ± standard error of the mean (*n* = 3). * *p* < 0.05, ** *p* < 0.01 versus 100 µM H_2_O_2_-alone challenged group. NF-κB, nuclear factor kappa-light-chain-enhancer; Nrf2, nuclear factor erythroid derived 2; SOD2, superoxide dismutase 2.

The Nrf2 pathway has been reported to be an important endogenous anti-oxidative signaling pathway [33]. It is also involved in the expression of antioxidant enzymes like SOD2 and catalase [34]. SOD2 is involved in the ROS defense system for intracellular antioxidants that rapidly detoxify superoxide (O_2_^−^) which produce H_2_O_2_ [35,36,37]. Catalase is an enzyme with a primary role related to managing H_2_O_2_ concentrations in human cells and converting H_2_O_2_ into H_2_O and O_2_ [38]. The levels of Nrf2, SOD2 and catalase were significantly increased following pretreatment with 50 nM of POM compared with the H_2_O_2_-exposed group (Figure 4a–c). To explore whether POM treatment could induce anti-inflammation, we investigated the protein expression of NF-κB and found that it was notably reduced in the POM-pretreated 50 nM group compared with the H_2_O_2_ alone challenged group (Figure 4d).

### 2.4. Pomalidomide Antagonized the Cytochrome c-Mediated Apoptotic Signaling Pathway After H_2_O_2_-Induced Cellular Death and Mitochondrial Function

**Figure 5:**
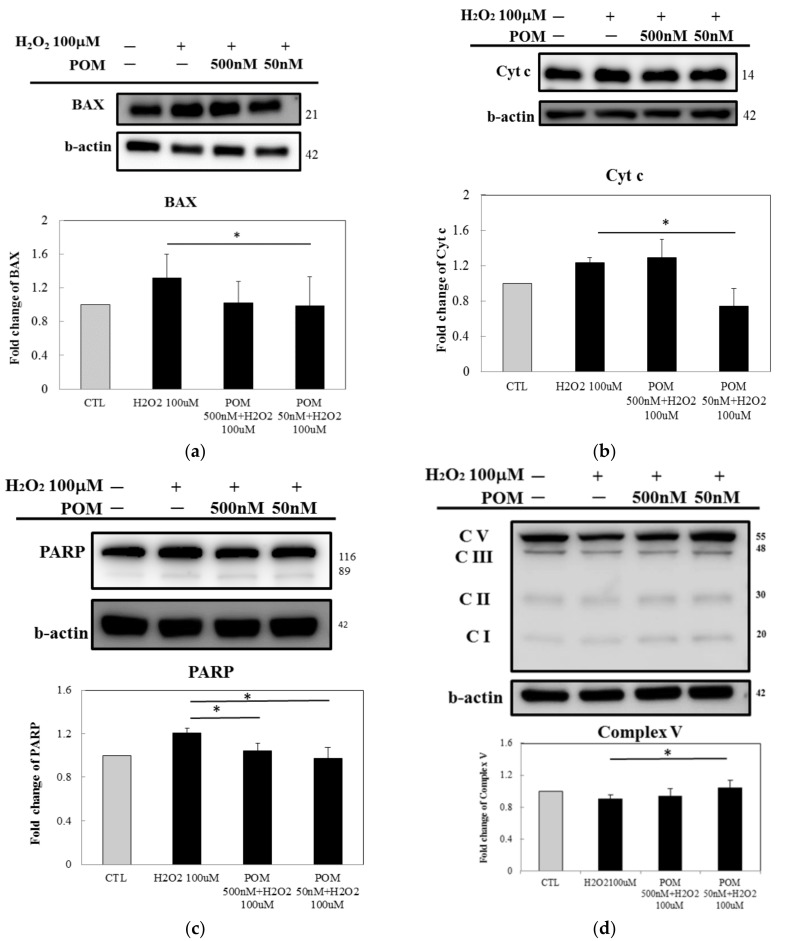
Pomalidomide (POM) decreases the expression of proteins within the mitochondrial Cytochrome c (Cyt c)-mediated apoptosis pathway and enhances the activity of mitochondria complex V. H_2_O_2_-induced cellular death raised protein levels of BAX, Cyt c and PARP within the apoptotic pathway (*p* ≤ 0.05 for control (CTL) versus H_2_O_2_ alone group). POM-pretreatment (particularly 50 nM) significantly inhibited the activation of the cell death; significantly inhibiting H_2_O_2_-induced BAX, Cyt c and PARP elevations (* *p* ≤ 0.05 versus 100 µM H_2_O_2_-alone challenged group). (**d**) The mitochondrial function after H_2_O_2_-induced oxidative stress, ATP production was mildly reduced by H_2_O_2_ insult. The Complex V (ATP synthase) was restored by POM-pretreatment (50 nM) group (* *p* ≤ 0.05 versus 100 µM H_2_O_2_-alone challenged group). Western blotting analysis was performed with antibodies specific for (**a**) BAX, (**b**) Cyt c, (**c**) PARP and (**d**) Complex V (evaluated by ATP5A antibody for ATP synthase). β-actin served as a control. Bars represent mean values ± standard error of the mean (*n* = 3). (Little effect of H_2_O_2_ challenge or POM treatment was evident across complexes I, II, III or IV (not shown)).

As a pro-apoptotic member of the Bcl-2 protein family, Bax promotes the release of Cytochrome c, thus inducing the activation of the cell death proteases, caspases [39]. In this study, pretreatment with POM (50 nM) suppressed the expression of Bax (Figure 5a). Cytochrome c has been shown to play a major role in the apoptotic signaling pathway. Once released, Cytochrome c is linked to the cytosolic protein Apaf-1 and causes the formation of apoptosomes [40]. Thus, we evaluated the intermediate proteins involved in the Cytochrome c-related apoptotic pathway using Western blot analysis. Our results indicated that POM-pretreatment (50 nM) reduced Cytochrome c levels (Figure 5b). In addition, the down-regulation of PARP was also inhibited (Figure 5c). We further investigated mitochondrial function after H_2_O_2_-induced oxidative stress using Western blot analysis and found that ATP-generated complex V was significantly greater in the POM (50 nM) pretreated groups than in the H_2_O_2_-insulted group (Figure 5d), despite no changes in the total ATP levels evaluated in Figure 2b.

## 3. Discussion

The pathophysiology of neurodegenerative disorders, whether acute as epitomized by stroke, or chronic as in AD, is complex and involves different vulnerable cell types across different brain regions responding to a diverse array of extrinsic and intrinsic challenges. Despite this, many of the main pathogenic mechanisms present in ischemia and AD as well as other acute and long-term neurodegenerations are common and include excitotoxic neurotransmitters, inflammatory pathways, oxidative damage, ionic imbalance and apoptosis [41,42,43,44,45,46,47,48]. The terminal results of acute ischemic cascades are neuronal death along with an irreversible loss of neuronal function. Thus, understanding the underlying mechanisms of neuronal death is important in developing new therapies for neurological disorders such as stroke or, indeed, any neurodegenerative disease [42,43,44,45,46,47,48,49,50,51]. Neuroprotective pharmacological therapies are extremely important approaches to preserve damaged brain regions. Previous studies have demonstrated that POM exerts a potent anti-inflammatory effect at significantly lower concentrations in vivo [20,21,22,23] and also alleviates neuronal apoptosis in traumatic brain injury [52,53]. Indeed, POM is involved in anti-inflammatory, anti-apoptotic and anti-oxidative processes that, particularly when combined, may represent viable therapeutic strategies in ischemic stroke and other neurodegenerative disorders.

To evaluate POM at a cellular level we used primary cortical neuronal cultures and demonstrated that POM mitigated H_2_O_2_-induced cytotoxicity and oxidative stress. Oxidative stress is characterized by the overproduction of ROS, which can injure the mitochondrial respiratory chain and mitochondrial defense systems [54]. Although treatment with POM decreased the production of H_2_O_2_, ATP production was mildly but not significantly reduced by H_2_O_2_ insult. A greater decline in ATP production following a similar H_2_O_2_ insult may have been evident in non-resting neurons (i.e., cells undergoing a physiological challenge), whereby their activation would have required a greater amount of ATP, compared to their resting state and would thus have amplified any oxidative stress-induced deficits in its production and potential mitigation by POM. It is additionally notable that ROS can affect cell constituents, such as DNA, proteins and lipids. DNA oxidation results in strand breaks and base modifications. Indeed, consistent with this concept, H_2_O_2_ elevated mitochondrial-specific superoxide generation and induced the formation of apoptotic bodies, as evaluated with the molecular probe MitoSox™ Red in combination with Hoechst 33342 blue fluorescence dye staining. Notably POM mitigated both and, thereby, reduced cell damage. Cerebral ischemia-evoked oxidative stress, inflammation and apoptosis eventually results in neuronal injury, dysfunction and death [13,41,54]. Therefore, this study investigated the potential effects of POM on the cellular defense mechanisms against oxidative stress and apoptosis signaling pathways.

During cerebral ischemia-reperfusion, NF-κB contributes to the generation of ROS and, particularly in microglia, activates NF-κB-driven inflammation [55,56]. This same inducible transcription factor plays a key role in mediating transient as well as sustained changes in gene expression in response to a diversity of external challenges, including oxidative stress. Indeed, in neurons NF-κB plays a role in promoting cellular survival as well as degenerative outcomes [54] and is considered a sensor of oxidative stress. In line with the literature [54], neurons exposed to H_2_O_2_, or induced to generate intracellular ROS, show potent NF-κB activation, associated with activation of genes coding for death or for protection, likely as a homeostatic response and the neuroprotection provided by POM mitigates this NF-κB-driven reaction. The Nrf2-mediated antioxidant protein, SOD2 detoxifies superoxide by transforming it to H_2_O_2_, which is then converted to H_2_O by catalase, glutathione peroxidase (GSHPx)/Glutathione Peroxidase 1 (Gpx1) [33,36].

The issues of positive controls for these types of in vitro cellular studies are important to briefly discuss. If POM had little or no effect, one would need to study agents with well documented anti-inflammatory/antioxidant properties to validate this system. However, given the clear efficacy of POM, the necessity of a positive control becomes less important. Notably, we have established the vulnerability of this and related cellular systems to oxidative and inflammatory stress and their mitigation in extensive prior studies [28,29,52,57]. Furthermore, we do not suggest that the use of H_2_O_2_ here reflects a role for H_2_O_2_ generation in stroke. We only used this molecule to generate ROS in a consistent quantitative manner in vitro. Needless to say, in both in vitro and in vivo models, ischemia (OGD, MCAo) also induces ROS and this is well established in the literature [58,59].

Our in vitro results demonstrated that SOD2, catalase and its upstream protein, Nrf2, were all significantly increased following POM pretreatment but the expression of NF-κB was suppressed. These changes thus confirm that POM exerted anti-oxidative and anti-inflammation effects. Moreover, ROS are directly implicated in oxidative impairment in ischemic tissues, thus resulting in cell death. In cellular models of H_2_O_2_-induced oxidative injury, mitochondria-mediated Cytochrome c release regulates the apoptotic pathway involving mediators such as BAX, Cytochrome c and PARP [60]. Activation of the release of BAX-Cytochrome c induces an apoptotic response. Our results revealed that POM diminished neuronal apoptosis via the suppression of the expression of BAX, Cytochrome c and its downstream protein PARP. This was evident at a POM concentration of 50 nM, which proved superior to that achieved at 500 nM, suggesting an inverted U-shaped dose-response curve.

It is important to note that the changes in Nrf2 and Complex V seen here do not establish the mechanism underpinning the protective actions of POM but are only correlational. In order to establish mechanisms, one would need to extend these studies by using specific antagonists, RNA silencing and/or null mutated animals. In a sense, the correlations here only test a null hypothesis. In other words, if NRF2 or Complex V did not change, it would suggest that these moieties are not involved in the POM mechanism.

The finding of our inverted “U” shaped dose/response cure for POM is not surprising. Most drugs have multiple actions and a neuroprotective effect at a lower dose is often reduced by other actions at higher doses. This is seen, for example, in neuroprotective activities in vitro with neurotrophic agents in both OGD and H_2_O_2_ challenge models [57,61]. The POM doses evaluated in our study are in line with those reported in the literature [20] and, more importantly, the POM 50 nM concentration is achievable in humans [25].

**Figure 6:**
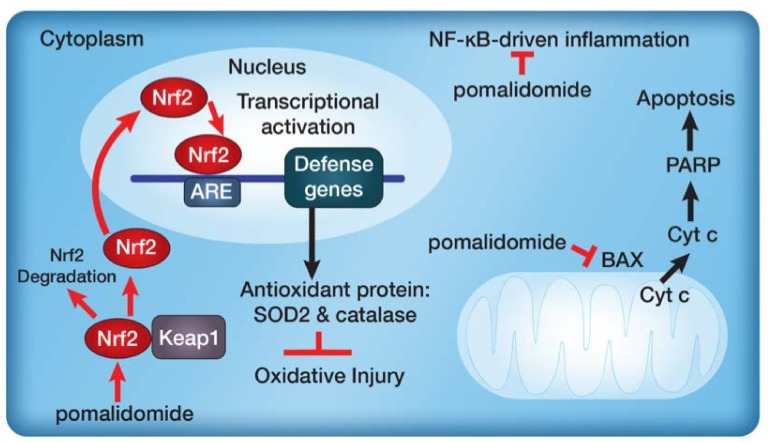
Proposed neuroprotective mechanisms underlying the cellular responses to H_2_O_2_-induced oxidative stress after pre-treatment with pomalidomide via Nrf2-SOD2/Catalase anti-oxidative signaling pathway and BAX-Cytochrome c (Cyt c)-PARP anti-apoptosis signaling pathway. Nrf2, nuclear factor erythroid derived 2; SOD2, superoxide dismutase 2.

In summary, cerebral ischemia and numerous other forms of injury in acute and chronic neurodegenerative conditions encompass several molecular mechanisms including oxidative stress, inflammation and programmed cell death [10,11,12,13,14,44,45,46,47,48,62]. These responses cause irreversible damage to cerebral tissues and even cell death. Here, we show that POM treatment exerts beneficial protective effects against H_2_O_2_-induced oxidative stress via its anti-apoptotic, anti-oxidative and anti-inflammatory properties (Figure 6). For anti-inflammation, POM inhibited ROS-related NF-κB-driven inflammatory expression and activated the production of Nrf2, thus inducing the production of the downstream antioxidants SOD2 and catalase. Finally, POM mitigated the BAX-mediated apoptosis signaling pathway through its downstream targets, exemplified by Cytochrome c and PARP.

## 4. Materials and Methods

### 4.1. Chemicals and Reagents

Pomalidomide was obtained from Selleckchem (Houston, TX, USA). Perdrogen™ 30% H_2_O_2_ (*w*/*w*), 1,3-[4,5-dimethyl-2-thiazolyl]-2,5-diphenyl-2-tetrazolium bromide (MTT), poly-l-lysine (molecular weight 70,000–150,000) and 2,3,5-triphenyltetrazolium chloride (TTC) were obtained from Sigma (St Louis, MO, USA). MitoSOX™ Red mitochondrial superoxide indicator, Hoechst 33342 (Trihydrochloride, Trihydrate), Pierce™ Coomassie (Bradford) Protein Assay Kit and standard culture reagents were acquired from Thermo Fisher Scientific (Waltham, MA, USA). CytoTox 96^®^ Non-Radioactive cytotoxic assay kit was obtained from Promega (Madison, WI, USA). Hydrogen peroxide assay and ATP assay kits were purchased from Biovision (Milpitas, California, USA). Total OXPHOS Rodent WB Antibody Cocktail and β-actin were acquired from Abcam (Cambridge, MA, USA), while SOD2 antibody was procured from LifeSpan Biosciences (Seattle, WA, USA). Nrf2, Catalase and NF-κB antibodies were obtained from Santa Cruz Biotechnology (Dallas, TX, USA). Cyt c and PARP were acquired from Cell Signaling Technology, Inc. (Danvers, MA, USA) and BAX from GeneTex, Inc. (Irvine, CA, USA).

### 4.2. Cell Cultures

Primary cultures of rat cortical neurons were prepared from the brains of Sprague-Dawley (SD) rat fetuses on embryonic day 17 (E17) to E18 (from BioLASCO Taiwan, Taipei, Taiwan). The neurons were cultured as described previously [52,63] with slight modifications. Briefly, embryonic cortices were digested with 0.05% trypsin-EDTA and 2 mg/mL of papain for 10 min at 37 °C. Subsequently, the samples were mechanically dissociated by gentle pipetting in Dulbecco’s modified Eagle’s medium supplemented with 5% *v*/*v* fetal bovine serum, 5% *v*/*v* horse serum, 0.6% *v*/*v* glucose, 0.5 mM glutamine, 1% penicillin/streptomycin/amphotericin B and 1% insulin-transferrin-sodium selenite media supplement. Cells were plated on poly-l-lysine coated culture dishes or plates. After 3 h of incubation, the cultured medium was replaced with a neurobasal medium supplemented with 0.5 mM glutamine, 2% B-27, 2% N-2 and 1% penicillin/streptomycin/amphotericin B. Thereafter, primary rat cortical neurons were incubated for 5–6 d in vitro prior to their use. The medium was changed every 3 d.

#### 4.2.1. Hydrogen Peroxide-Induced Injury and Pomalidomide Pretreatment

After 5–6 d in vitro, primary cortical neurons were pretreated with 500 nM or 50 nM POM for 24 h. The lower of these doses, in particular, has relevance to concentrations achievable in humans following routine dosing [20,25]. Following this POM pretreatment, cells were challenged with 100 µM H_2_O_2_ for 0.5 h, then washed with PBS and cultured in fresh media without POM and H_2_O_2_.

#### 4.2.2. Cell Viability

Primary cortical neurons were plated in 24-well plates at a density of 5 × 10^5^ cells/well and cultured for 5 d. After POM pretreatment and H_2_O_2_-exposure, the MTT reagent was added to the culture medium in each well at a final concentration of 0.5 mg/mL. After incubating the mixture for 1.5 h at 37 °C, sodium dodecyl sulfide was added to dissolve the formazan crystals and the material was subsequently transferred to a 96-well plate. The absorbance was measured at a wavelength of 590 nm using a microplate reader (TECAN, Männedorf, Switzerland).

In parallel studies to detect cell death, lactate dehydrogenase (LDH) in the culture medium and total LDH after cell lysis were measured using a Promega cytotoxicity detection kit according to the manufacturer′s instructions. LDH release was represented as percentage of LDH released in the culture medium to total LDH (medium and lysates) and was measured at 490 nm using a microplate reader.

### 4.3. The H_2_O_2_ Production Assay

To detect H_2_O_2_ production, primary cortical neurons were cultured in 24-well plates for 5 d. After POM pretreatment and H_2_O_2_-exposure, cells were washed with HBSS and equal volumes of lysates were quantified with red fluorescence (Ex/Em = 535/587 nm) using a Hydrogen Peroxide Colorimetric/Fluorometric Assay kit (BioVision) according to the manufacturer’s instructions.

### 4.4. Quantification of ATP

For the ATP assay, primary cortical neurons were plated in 24-well plates at a density of 5 × 10^5^ cells/well and cultured for 5 d. After POM pretreatment and H_2_O_2_-challenge, ATP levels in equal volumes of cell lysates were quantified with red-fluorescent (Ex/Em = 535/587 nm) using an ATP Colorimetric/Fluorometric Assay kit (BioVision) according to the manufacturer’s instructions.

### 4.5. Western Blot Analysis

Cells were lysed in radioimmunoprecipitation assay (RIPA) lysis buffer containing 1% protease inhibitor cocktail. Subsequently, total protein was extracted and the protein samples were quantified using a Pierce™ Coomassie (Bradford) Protein Assay Kit. The protein samples were separated using SDS-PAGE and were subsequently transferred onto poly-vinylidene fluoride membranes and blocked with 5% non-fat milk for 1 h at room temperature. Membranes were incubated at 4 °C overnight with primary antibodies, followed by incubations with the corresponding secondary antibodies for 1 h at room temperature. The obtained images were scanned using ChemiDoc (Bio-rad, Philadelphia, PA, USA) and the resulting data were analyzed using Image J software; β-actin was used as a loading control. All images were evaluated in compliance with the digital image and integrity policies defined in www.nature.com/srep/policies/index.html#digital-image.

### 4.6. Determination of Cellular Reactive Oxygen Species (ROS) Production

Hoechst 33342 staining was used to observe morphological changes of primary cortical neurons exposed to POM pretreatment and H_2_O_2_ challenge. MitoSOX™ Red (Molecular Probes, Invitrogen, CA, USA) was the mitochondrial superoxide indicator used in live cells and superoxide produced a red fluorescence. The live cells were incubated with 5 µM of MitoSOX™ Red and 5 µg/mL of Hoechst 33342 (Molecular Probes, Invitrogen, CA, USA). After washing three times in Hank’s Balanced Salt Solution, the stained cells were observed using fluorescence microscopy (Nikon Eclipse Ti-U). Cells with fragmented or condensed DNA were determined as apoptotic cells by using image J software in order to evaluate whether or not POM protects against H_2_O_2_-induced apoptosis. The Hoechst 33342 fluorescent nuclear stain, apoptosis quantitative analyses are the percentage of apoptotic cells and was calculated as the ratio of apoptotic cells to the total cells counted. The fluorescence intensity of MitoSOX™ Red was determined using image J software. For the assessment of intracellular mitochondrial superoxide production, MitoSOX™ Red fluorescent stain, the fluorescence intensity was quantified and normalized to number of cells present and then compared in relation to fold change with the control group (=1).

### 4.7. Statistical Analysis

All data are expressed as the mean ± standard error of the mean (SEM) values. Comparisons among different groups were performed using one-way analysis of variance followed by Dunnett tests. Differences were considered statistically significant at *p* < 0.05.
